# Age Difference in the Connection Between Systemic Inflammatory Response and Metabolic Syndrome

**DOI:** 10.1210/clinem/dgae669

**Published:** 2024-09-25

**Authors:** Haishan Wei, Dan Xu, Jiying Chen, Haiyan Yu, Xiaodong Zhang, Zhiyun Liu, Chen Liu, Yuan Guo

**Affiliations:** Department of General Practice, Qilu Hospital of Shandong University, Jinan, 250012, China; Department of General Practice, Qilu Hospital of Shandong University, Jinan, 250012, China; Department of General Practice, Qilu Hospital of Shandong University, Jinan, 250012, China; School of General Practice and Continuing Education, Capital Medical University, Beijing, 100000, China; Department of General Practice, Qilu Hospital of Shandong University, Jinan, 250012, China; Department of General Practice, Qilu Hospital of Shandong University, Jinan, 250012, China; Department of General Practice, Qilu Hospital of Shandong University, Jinan, 250012, China; Department of General Practice, Qilu Hospital of Shandong University, Jinan, 250012, China

**Keywords:** systemic immune-inflammation index, metabolic syndrome, national health and nutrition examination survey, age differences

## Abstract

**Background:**

This research aims to investigate the connection between systemic inflammatory response and metabolic syndrome (MetS) across different age groups, with the aim of proposing more targeted recommendations.

**Methods:**

This study enrolled 15 959 adults from the 2001-2018 National Health and Nutrition Examination Survey of whom 6739 were diagnosed with MetS. After dividing the systemic immune-inflammation index (SII) into 4 quartiles, the Kruskal–Wallis test and weighted chi-square test were employed to assess statistical differences. Weighted multivariable logistic regression analysis, subgroup analysis, sensitivity analysis, and restricted cubic spline were employed to examine the relationship between SII and MetS.

**Results:**

Our study revealed that SII exhibits a quantitative association with MetS [odds ratio (OR) = 1.56; 95% confidence interval (CI): 1.37-1.79; *P* < .001]. Elevated SII is an independent risk factor for the 5 components of MetS. Different age groups and alcohol consumption status could modify the connection between SII and MetS. This connection was statistically significant in the 18 to 65 age group but not in the elderly subgroup (OR = 1.08; 95% CI, .95-1.23; *P* = .248). Multiple imputation confirmed the robustness of our results. Moreover, the connection exhibits an inverted U-shaped curve.

**Conclusion:**

Our research highlights the predictive significance of SII in forecasting the incidence of MetS in young and middle-aged populations. The differences in inflammatory mechanisms across various age groups necessitate further research for exploration.

Influenced by modern lifestyles and eating habits, the incidence of metabolic syndrome (MetS) has exhibited a gradual upward trend over the past several years (1), becoming the largest burden of noncommunicable diseases globally (2). Notably, the incidence rate increased rapidly in the younger population (3). Although a considerable volume of reaserch confirms the association between MetS and a proinflammatory state, no study to date has validated the age-related differences. MetS refers to a syndrome including dyslipidemia, central obesity, hyperglycemia, and hypertension (4). This metabolic disorder can lead to multisystem consequences, with the most significant clinical impacts being the high incidence and mortality rates of cardiovascular disease events (5).

The occurrence of acute inflammation is typically associated with tissue damage repair. By contrast, chronic inflammation represents a long-term, disordered, and poorly adaptive regulatory response, encompassing inflammation activation and tissue remodeling (6). Currently, 2 prevailing theories elucidate the mechanisms behind persistent chronic inflammation. One suggests that prolonged viral infections lead to the accumulation of inflammatory cells. The other posits that cellular debris and mislocated and/or misfolded self-molecules may play a dominant role (7). Both mechanisms are intricately linked to aging. The culmination of the inflammatory response involves resolving the induced inflammation and restoring homeostasis within the body. Insufficient resolution of inflammation or failure to reestablish balance can lead to chronic inflammation or autoimmunity (8).

Considering that MetS is characterized by a cluster of risk factors for cardiovascular and metabolic diseases, insights from prior research into cardiovascular risk factors could enhance our comprehension of the clinical onset and progression of inflammation in MetS. Numerous studies have established the link between inflammatory markers and cardiovascular risk factors (9-11). Similarly, elevated levels of inflammatory markers have also been confirmed to correlate with insulin resistance and diabetes (12-14). In patients with heart failure, increased inflammatory markers have also been shown to relate to the severity of the disease and poorer clinical outcomes (15, 16). Emerging research expanding on the relationship between the systemic immune-inflammation index (SII) and nonneoplastic diseases has illustrated its association with increased urinary albumin excretion (17) and hepatic steatosis (18), advocating for its adoption as a novel biomarker for cardiovascular risk factors (19). Chronic inflammation is closely associated not only with cardiovascular and metabolic diseases but has also been demonstrated to correlate with increased incidence rates of multiple diseases. Research findings support that this encompasses osteoporosis (20-22), cognitive decline and dementia (23-25), frailty and disability (26, 27) as well as cancer (28, 29). Considering the simplicity and accessibility of the measurement of SII and the diagnosis of MetS in community settings, we recommend including this indicator in early community screening protocols for cardiovascular disease risk factors.

Introduced by Hu et al in 2014, the SII is originally aimed at quantifying inflammation levels to predict postoperative outcomes in cancer patients (30). The SII has since been acknowledged for its precision in assessing inflammatory states (31). Existing studies have shown that the SII is consistent with increased urinary albumin excretion (17) and hepatic steatosis (18), and it has been suggested as a biomarker for cardiovascular risk factors (19). Notably, the significant predictive accuracy, noninvasiveness, easy accessibility, and cost-effectiveness of the SII substantially augment its practical application in clinical settings (18).

Our research aims to investigate the correlation between the SII and the incidence of MetS, with an emphasis on age-related variations. Early identification, diagnosis, and intervention can help to reduce the incidence of MetS and its complications. The healthcare burden caused by MetS and its complications is substantial. With the focus of global healthcare reform gradually shifting from reducing mortality from cardiovascular events to preventing noncommunicable diseases, we call on community doctors to conduct universal screenings for high-risk groups and opportunistic screenings for medium- to low-risk groups in their daily practice. Given the connection between elevated inflammatory markers and a higher risk of poor disease prognosis, we recommend regular monitoring of inflammatory indicators in individuals diagnosed with MetS, complemented by lifestyle modifications and pharmacological interventions as required for individuals exhibiting elevated inflammation levels.

## Materials and Methods

### Study Design and Population

The National Health and Nutrition Examination Survey (NHANES) program combines interviews and physical examinations to assess the nutrition and health conditions of the US population. This initiative is overseen by the Centers for Disease Control and Prevention and is refreshed biennially. The Institutional Review Board of the National Center for Health Statistics has approved all protocols, and informed consent from participants is mandatory (32). The data for this research is accessible through the official website (https://wwwn.cdc.gov/nchs/nhanes/default.aspx).

**Figure 1.**
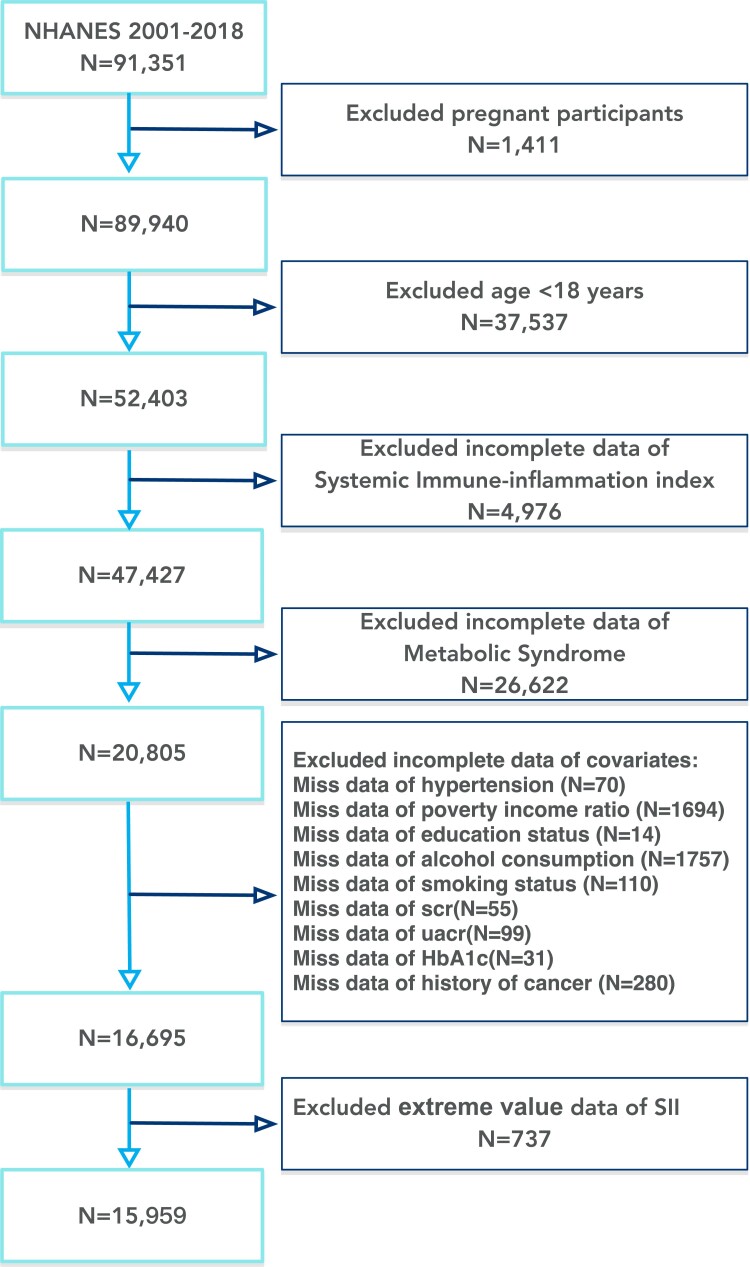
This figure displays the recruitment process for NHANES 2001-2018 participants, outlining the exclusion criteria and the final number of participants. Abbreviations: NHANES, National Health and Nutrition Examination Survey.

We excluded participants who were pregnant, were under the age of 18, or had incomplete critical data or confounding factors (including sex, age, poverty income ratio (PIR), waist circumference (WC), systolic blood pressure, diastolic blood pressure, fasting glucose (FPG), total cholesterol, triglycerides (TG), high-density lipoprotein cholesterol (HDL-C), serum creatinine (Scr), urine albumin to creatinine ratio, hemoglobin A1c, SII, race, education level, smoking status, alcohol consumption, MetS, history of hypertension). Upon reviewing the extreme values of the SII data, they were determined not to be outliers or errors. However, to ensure the stability and representativeness of the data, extreme values were still removed. The specific method involved removing data where SII < Q1—1.5 × the interquartile range and SII > Q3 + 1.5 × interquartile range. This study ultimately included 15 959 participants for complete data analysis. Figure 1 illustrates the process of sample selection.

### MetS

The criteria for diagnosing MetS are derived from the revised guidelines of the National Cholesterol Education Program's Adult Treatment Panel III guidelines (33). The criteria are as follows: (1) elevated TG ≥ 1.7 mmol/L; (2) decreased HDL-C: men <1.03 mmol/L, women <1.29 mmol/L; (3) FPG ≥ 5.6 mmol/L; (4) increased WC: men >102 cm, women >88 cm; (5) hypertension: blood pressure ≥130/85 mmHg or currently taking antihypertensive medication.

At the initial stage, blood samples were obtained after participants had fasted for a minimum of 8 hours to measure the levels of the observed indicators. Additionally, data on blood pressure and WC were gathered during a physical examination at the mobile testing center.

### SII

The individuals were categorized into 4 groups based on the quartiles of the SII (Q1, Q2, Q3, Q4), using the first quartile (Q1) as the reference group. According to Professor Hu, the calculation formula is as follows (34):


$$\mathrm{SII}\;(\times 10^{3}\mathrm{cells}/\mu l)=\;\frac{\mathrm{platelet}\;\mathrm{count}\;\times \;\mathrm{neutrophil}\;\mathrm{count}}{\mathrm{lymphocyte}\;\mathrm{count}}$$

### Covariates

Utilizing a computer-assisted personal interviewing system, NHANES collects sociodemographic data and health-related factors during household interviews. The information gathered includes age, sex, race, education, PIR, smoking, alcohol consumption, and disease status. Age is categorized as 18 to 30, 31 to 45, 46 to 65, or >65 years. Racial/ethnic categories include Mexican American, other Hispanic, non-Hispanic White, non-Hispanic Black, and other race. Educational level is classified as less than high school, high school or equivalent, or more than high school. The PIR is divided into ≤1 (low income level), >1 to <4 (middle income level), and ≥4 (high income level). Smoking status is recorded as never smokers, former smokers, or current smokers according to the glossary of the NHANES. Similarly, drinking status is divided into lifetime abstainers, former drinkers, and current drinkers.

### Statistical Analysis

Given the intricate sampling framework of NHANES, our analyses comprehensively included the sample weights, clustering, and stratification as advised by the Centers for Disease Control and Prevention, a crucial step for the accurate analysis of NHANES data. Sample weights were calculated in the following manner: dividing the fasting subsample 2-year Mobile Examination Center weight by 9. The data is expressed as means with SEs for continuous variables and as frequencies and percentages for categorical variables. To assess statistical differences, the Kruskal–Wallis test was applied for continuous variables, and weighted chi-square tests were conducted for categorical variables.

Associations between the 4 groups were studied using weighted multivariate logistic regression. The crude model did not adjust for covariates. Model 1 was adjusted for sex, age, and race. Model 2 was adjusted for sex, age, race, education, PIR, smoking, alcohol consumption, Scr, urine albumin to creatinine ratio, and glycated hemoglobin. Subgroup analyses of the association were performed using stratification factors. To assess the heterogeneity of the association across subgroups, we introduced an interaction term test and calculated *P*-values using the likelihood ratio test to compare two models. Sensitivity analyses were performed using multiple imputation to address missing data for PIR, educational level, alcohol consumption status, smoking status, serum creatinine, urine albumin to creatinine ratio, glycated hemoglobin, history of hypertension, and cancer. The multiple imputation by chained equations method was utilized to impute the missing data.

A restricted cubic spline (RCS) analysis was employed to investigate the nonlinear associations, as well as its individual components across various age cohorts. For the analysis, the SII was logarithmically transformed due to its right-skewed distribution. Data analysis and figure generation in this study were conducted using Stata 17 and R software (version 4.3.2).

## Results

### Baseline Characteristics of General Clinical Data

This study sample comprised 15 959 participants, including 8188 males and 7771 females averaging an age of 47.25 ± 0.24 years. Table 1 presents the characteristics of individuals grouped by SII quartiles. MetS was diagnosed in 42.48% of the individuals. Individuals were divided into quartiles, distributed as follows: Q1 group, 2 < SII < 321; Q2 group, 321 < SII < 446; Q3 group, 446 < SII < 620 and Q4 group, 620 < SII < 1136. Significant differences were identified among the 4 SII groups regarding age, sex, race, PIR, WC, systolic blood pressure, FPG, TC, TG, HDL-C, Scr, urine albumin to creatinine ratio, glycosylated hemoglobin, SII, education, smoking, alcohol consumption, and hypertension (all *P-*values < .05). Consistent with the trend of increasing SII values, the demographic and biochemical characteristics included being male, older age, higher WC, increased systolic blood pressure, elevated FPG, increased TG, lower HDL-C, elevated urine albumin to creatinine ratio, increased glycated hemoglobin, non-Hispanic White, and current smokers and having MetS. Conversely, characteristics associated with lower SII values included being female, non-Hispanic Black, other races, and never smokers and not having MetS.

**Table 1.** Baseline characteristics of the participants

	Total (n = 15 959)	Q1 (2 < SII < 321)	Q2 (321 < SII < 446)	Q3 (446 < SII < 620)	Q4 (620 < SII < 1136)	*P*-value
Sex, n (%)						<.001
Male	8188 (51.31)	2259 (56.62)	2142 (53.68)	1961 (49.10)	1826 (45.82)	
Female	7771 (48.69)	1731 (43.38)	1848 (46.32)	2033 (50.90)	2159 (54.18)	
Age (years)	47.25 ± 0.24	46.21 ± 0.41	46.29 ± 0.37	47.66 ± 0.36	48.56 ± 0.37	<.001
PIR	3.06 ± 0.03	2.99 ± 0.04	3.15 ± 0.04	3.11 ± 0.04	2.99 ± 0.05	.007
WC (cm)	98.80 ± 0.22	96.07 ± 0.33	97.75 ± 0.36	99.65 ± 0.33	101.27 ± 0.36	<.001
SBP (mmHg)	121.58 ± 0.20	120.60 ± 0.36	121.27 ± 0.31	121.44 ± 0.36	122.80 ± 0.32	<.001
DBP (mmHg)	70.52 ± 0.18	70.02 ± 0.25	70.69 ± 0.24	70.51 ± 0.28	70.84 ± 0.26	.342
FBG (mmol/L)	5.53 ± 0.02	5.47 ± 0.03	5.52 ± 0.03	5.53 ± 0.03	5.59 ± 0.03	.002
TC (mmol/L)	5.04 ± 0.01	4.98 ± 0.03	5.06 ± 0.02	5.07 ± 0.02	5.03 ± 0.02	.008
TG (mmol/L)	1.48 ± 0.01	1.41 ± 0.03	1.45 ± 0.02	1.51 ± 0.03	1.52 ± 0.02	<.001
HDL-C (mmol/L)	1.39 ± 0.01	1.42 ± 0.01	1.40 ± 0.01	1.38 ± 0.01	1.37 ± 0.01	<.001
Scr (umol/L)	78.20 ± 0.25	79.09 ± 0.40	78.53 ± 0.49	77.52 ± 0.43	77.65 ± 0.49	<.001
Uacr (mg/g)	27.50 ± 1.63	20.93 ± 1.82	23.08 ± 2.74	26.55 ± 2.83	38.09 ± 4.58	<.001
HbA1c	5.58 ± 0.01	5.56 ± 0.02	5.56 ± 0.02	5.57 ± 0.02	5.63 ± 0.02	.022
SII (×10^9/L)	495.74 ± 2.79	244.30 ± 1.23	383.80 ± 0.67	525.55 ± 1.00	793.03 ± 2.83	<.001
Race, n (%)						<.001
Mexican American	2658 (16.66)	601 (15.06)	738 (18.50)	679 (17.00)	640 (16.06)	
Other Hispanic Race	1289 (8.08)	295 (7.39)	344 (8.62)	348 (8.71)	302 (7.58)	
Non-Hispanic White	7464 (46.77)	1421 (35.61)	1833 (45.94)	1993 (49.90)	2217 (55.63)	
Non-Hispanic Black	3107 (19.47)	1216 (30.48)	710 (17.79)	649 (16.25)	532 (13.35)	
Other race	1441 (9.03)	457 (11.45)	365 (9.15)	325 (8.14)	294 (7.38)	
Education, n (%)						.002
Less than high school Grad	3899 (24.43)	953 (23.88)	983 (24.64)	1008 (25.24)	955 (23.96)	
High school grad/GED or equivalent	3674 (23.02)	911 (22.83)	872 (21.85)	898 (22.48)	993 (24.92)	
More than high school grad	8386 (52.55)	2126 (53.28)	2135 (53.51)	2088 (52.28)	2037 (51.12)	
Smok, n (%)						<.001
Never smoker	8541 (53.52)	2281 (57.17)	2199 (55.11)	2136 (53.48)	1925 (48.31)	
Current smoker	3310 (20.74)	740 (18.55)	750 (18.80)	826 (20.68)	994 (24.94)	
Former smoker	4108 (25.74)	969 (24.29)	1041 (26.09)	1032 (25.84)	1066 (26.75)	
Alcohol consumption, n (%)						<.001
Lifetime abstainer	2079 (13.03)	547 (13.71)	512 (12.83)	536 (13.42)	484 (12.15)	
Current drinker	10 890 (68.24)	2724 (68.27)	2776 (69.57)	2717 (68.03)	2673 (67.08)	
Former drinker	2990 (18.74)	719 (18.02)	702 (17.59)	741 (18.55)	828 (20.78)	
MetS, n (%)						<.001
Yes	6779 (42.48)	1517 (38.02)	1682 (42.16)	1705 (42.69)	1875 (47.05)	
No	9180 (57.52)	2473 (61.98)	2308 (57.84)	2289 (57.31)	2110 (52.95)	
Hypertension, n (%)						<.001
Yes	6769 (42.41)	1628 (40.80)	1569 (39.32)	1698 (42.51)	1874 (47.03)	
No	9190 (57.59)	2362 (59.20)	2421 (60.68)	2296 (57.49)	2111 (52.97)	

Abbreviations: DBP, diastolic blood pressure; FBG, fasting glucose; HbA1c, glycated hemoglobin; HDL-C, high-density lipoprotein cholesterol; MetS, metabolic syndrome; PIR, poverty income ratio; SBP, systolic blood pressure; Scr, serum creatinine; SII, systemic immune-inflammation index; TC, total cholesterol; TG, triglyceride; Uacr, urine albumin to creatinine ratio; WC, waist circumference.

### Association Between SII and MetS

Presented in Table 2 are the outcomes of the weighted multivariable logistic regression analysis, examining the relationship between SII and the occurrence of MetS and its individual components. This relationship was consistently significant across all three models: Crude model [odds ratio (OR) = 1.27; 95% confidence interval (CI), 1.20-1.35; *P* < .001], model 1 (OR = 1.26; 95% CI, 1.19-1.34; *P* < .001), and model 2 (OR = 1.25; 95% CI, 1.17-1.33; *P* < .001). This indicates that for every unit increase in the log2-SII score, there is a 25% higher probability of developing MetS. It also revealed that MetS prevalence rises with increasing SII quartiles. In comparison to the Q1, subjects in the Q2 and Q3 exhibited higher risks of MetS, with the risk increasing by 36% (OR = 1.36; 95% CI, 1.18-1.57; *P* < .001) and 29% (OR = 1.29; 95% CI, 1.13-1.48; *P* < .001), respectively, both statistically significant. Consistent with this, those in Q4 demonstrated a 56% higher risk of MetS, which was also statistically significant (OR = 1.56; 95% CI, 1.37-1.79; *P* < .001). Unlike the unadjusted crude model, the adjusted prevalence rates for the Q2 and Q3 groups did not correspond with the theoretical expectation of higher inflammation levels leading to higher disease rates, potentially indicating that the confounding factors may play different roles at moderate-low and moderate-high inflammation levels. This change was observed both in model 1 and model 2, suggesting that these confounding factors involve elements such as age, sex, and ethnicity. Further exploration and confirmation are warranted.

**Table 2.** Association of SII with MetS and its components

	Crude model			Model 1			Model 2		
	OR	(95% CI)	*P*-value	OR	(95%CI)	*P-*value	OR	(95% CI)	*P-*value
MetS			<.001			<.001			<.001
Log-SII	1.27	(1.20, 1.35)	<.001	1.26	(1.19, 1.34)	<.001	1.25	(1.17, 1.33)	<.001
Q1	Ref.			Ref.			Ref.		
Q2	1.25	(1.11, 1.41)	<.001	1.32	(1.15, 1.51)	<.001	1.36	(1.18, 1.57)	<.001
Q3	1.28	(1.14, 1.44)	<.001	1.25	(1.10, 1.43)	.001	1.29	(1.13, 1.48)	<.001
Q4	1.61	(1.43, 1.80)	<.001	1.58	(1.39, 1.79)	<.001	1.56	(1.37, 1.79)	<.001
Elevated FPG			<.001			<.001			<.001
Log-SII	1.14	(1.07, 1.22)	<.001	1.13	(1.06, 1.22)	<.001	1.12	(1.02, 1.22)	.015
Q1	Ref.			Ref.			Ref.		
Q2	0.95	(0.85, 1.06)	.365	0.95	(0.84, 1.08)	.448	0.93	(0.81, 1.08)	.340
Q3	1.11	(0.99, 1.25)	.068	1.09	(0.96, 1.24)	.176	1.13	(0.96, 1.32)	.140
Q4	1.24	(1.10, 1.39)	<.001	1.19	(1.05, 1.35)	.005	1.16	(0.98, 1.37)	.076
Low HDL-C			<.001			<.001			<.001
Log-SII	1.21	(1.13, 1.30)	<.001	1.17	(1.09, 1.25)	<.001	1.16	(1.08, 1.24)	<.001
Q1	Ref.			Ref.			Ref.		
Q2	1.22	(1.10, 1.37)	<.001	1.22	(1.09, 1.37)	.001	1.25	(1.11, 1.40)	<.001
Q3	1.31	(1.18, 1.46)	<.001	1.26	(1.13, 1.41)	<.001	1.29	(1.15, 1.44)	<.001
Q4	1.45	(1.27, 1.66)	<.001	1.36	(1.19, 1.56)	<.001	1.34	(1.16, 1.54)	<.001
Elevated TG			<.001			<.001			<.001
Log-SII	1.17	(1.10, 1.24)	<.001	1.17	(1.10, 1.25)	<.001	1.15	(1.08, 1.23)	<.001
Q1	Ref.			Ref.			Ref.		
Q2	1.19	(1.06, 1.33)	.003	1.23	(1.08, 1.39)	.002	1.23	(1.08, 1.40)	.002
Q3	1.21	(1.09, 1.35)	.001	1.20	(1.07, 1.36)	.003	1.21	(1.07, 1.36)	.002
Q4	1.34	(1.19, 1.50)	<.001	1.32	(1.17, 1.50)	<.001	1.29	(1.14, 1.47)	<.001
Elevated WC			<.001			<.001			<.001
Log-SII	1.42	(1.33, 1.51)	<.001	1.34	(1.26, 1.43)	<.001	1.33	(1.25, 1.42)	<.001
Q1	Ref.			Ref.			Ref.		
Q2	1.34	(1.19, 1.52)	<.001	1.34	(1.18, 1.53)	<.001	1.37	(1.20, 1.57)	<.001
Q3	1.59	(1.42, 1.78)	<.001	1.51	(1.34, 1.70)	<.001	1.53	(1.36, 1.73)	<.001
Q4	1.92	(1.70, 2.17)	<.001	1.71	(1.51, 1.94)	<.001	1.70	(1.50, 1.93)	<.001
Elevated BP			<.001			<.001			<.001
Log-SII	1.18	(1.10, 1.26)	<.001	1.19	(1.11, 1.28)	<.001	1.18	(1.10, 1.27)	<.001
Q1	Ref.			Ref.			Ref.		
Q2	1.03	(0.91, 1.16)	.620	1.08	(0.93, 1.24)	.317	1.10	(0.95, 1.27)	.208
Q3	1.17	(1.04, 1.32)	.012	1.16	(1.00, 1.35)	.055	1.18	(1.01, 1.38)	.035
Q4	1.47	(1.29, 1.68)	<.001	1.50	(1.28, 1.75)	<.001	1.48	(1.27, 1.73)	<.001

The crude model did not adjust for covariates. Model 1 was adjusted for sex, age, and race. Model 2 was adjusted for sex, age, race, educational level, poverty income ratio, smoking status, alcohol consumption, serum creatinine, urine albumin to creatinine ratio, and glycated hemoglobin.

Abbreviations: BP, blood pressure; CI, confidence interval; HDL-C, high-density lipoprotein cholesterol; MetS, metabolic syndrome; OR, odds ratio; SII, systemic immune-inflammation index; TG, triglyceride; WC, waist circumference.

It was clear after correcting for confounding variables that the SII was still substantially linked to the start of MetS whether it was classified as a continuous or categorical variable: hypertriglyceridemia (OR = 1.15; 95% CI, 1.08-1.23; *P* < .001), increased WC (OR = 1.33; 95% CI, 1.25-1.42; *P* < .001), and decreased HDL-C (OR = 1.16; 95% CI, 1.08-1.24; *P* < .001). However, SII was shown to have a statistically significant positive connection with high FPG only when taken into account as a continuous variable (OR = 1.12; 95% CI, 1.02-1.22; *P* = .015). In the elevated blood pressure group, only continuous SII (OR = 1.18; 95% CI, 1.10-1.27; *P* < .001) and the highest quartile of SII levels (Q4) (OR = 1.48; 95% CI, 1.27-1.73; *P* < .001) had significant statistical relevance. Among all components of MetS, it was discovered that the likelihood of elevated inflammatory levels was highest for a rise in WC. An increased risk of developing MetS components was consistently linked to an upward trend in SII levels.

Considering the sex differences in MetS and its components, we examined the associations stratified by sex (Supplementary Table S1, Supplementary Table S2) (35). Our results showed that the continuous variable log-SII was statistically significant for MetS in both men and women, with a notably higher risk observed in women (OR = 1.38; 95% CI, 1.25-1.53; *P* < .001) compared to men (OR = 1.16; 95% CI, 1.06-1.27; *P* = .002). Among the 5 components, women exhibited a higher risk than men across all components. In men, only the component of elevated WC showed a statistically significant association with both the continuous variable log-SII and the categorical variable.

### Subgroup Analysis

**Figure 2.**
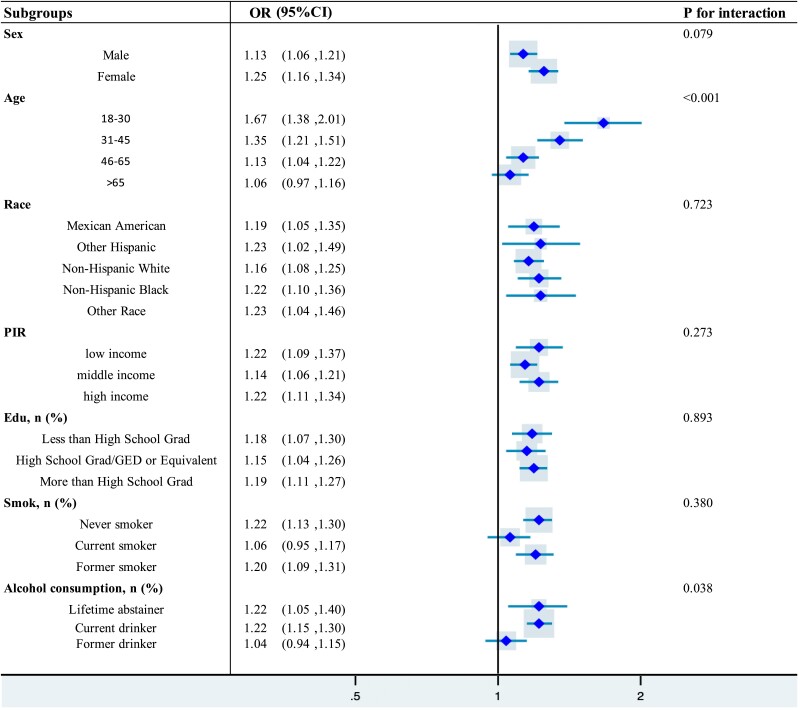
This figure presents the interaction analysis between SII and MetS across different subgroups, with significant differences indicated by *P*-values and confidence intervals. Abbreviations: MetS, metabolic syndrome; SSI, systemic immune-inflammation index.

Figure 2 demonstrates the link between SII and MetS as identified through subgroup analysis. This connection was more pronounced and statistically significant within subgroups of females, individuals aged 18 to 30, other Hispanic ethnicities, those with an education level of high school or above, low-income families, never smokers, and never drinkers. Despite not being statistically significant, a consistent relationship between the SII and MetS was seen in individuals over 65, current smokers, and past drinkers. Interaction tests indicated that the relationship was significantly affected by stratifications of age and drinking status (interaction *P*-values < .05, as shown in Fig. 2).

### Comparing Age Group Differences

Table 3 demonstrates in the age-stratified analysis, it becomes evident that the increase in the SII is more predictive of the risk of developing MetS among younger individuals compared to older adults. Specifically, in the population aged 18 to 45 years, higher SII categories consistently suggest a higher risk of developing MetS in the presence of heightened inflammatory levels. However, in the population over 45 years, although a continuous steady connection between increased SII and MetS is observed, this connection does not uniformly translate into increased risk with higher levels of inflammation in the Q2 and Q3 categories. Instead, a decline in risk is noted, suggesting the need for further experimental studies to explore the differences in inflammation levels and their impact on disease incidence among middle-aged populations. In older persons, there is no statistical significance between the SII and MetS risk, which indicates that the SII may not be a reliable predictor of disease risk in this demographic.

**Table 3.** Association of SII with MetS in different age groups

MetS	Crude model			Model 1			Model 2		
	OR	(95% CI)	*P-*value	OR	(95% CI)	*P-*value	OR	(95% CI)	*P-*value
18-30			**.021**			**<.001**			**<.001**
Continous Log-SII	1.51	(1.16, 1.97)	**.002**	1.51	(1.16, 1.96)	**.003**	1.47	(1.13, 1.90)	**.005**
Quartile 1	Ref.			Ref.			Ref.		
Quartile 2	1.56	(0.97, 2.49)	.064	1.55	(0.98, 2.46)	.060	1.63	(1.03, 2.59)	**.037**
Quartile 3	1.82	(1.15, 2.89)	**.011**	1.76	(1.11, 2.80)	**.017**	1.83	(1.15, 2.91)	**.011**
Quartile 4	2.22	(1.32, 3.74)	**.003**	2.21	(1.32, 3.71)	**.003**	2.15	(1.28, 3.62)	**.004**
31-45			**<.001**			**<.001**			**<.001**
Continous Log-SII	1.39	(1.22, 1.59)	**<.001**	1.45	(1.27, 1.65)	**<.001**	1.44	(1.26, 1.65)	**<.001**
Quartile 1	Ref.			Ref.			Ref.		
Quartile 2	1.20	(0.95, 1.52)	.123	1.19	(0.94, 1.50)	.148	1.34	(1.05, 1.71)	**.017**
Quartile 3	1.22	(0.96, 1.55)	.099	1.24	(0.98, 1.57)	.079	1.36	(1.06, 1.73)	**.015**
Quartile 4	1.70	(1.35, 2.14)	**<.001**	1.82	(1.45, 2.29)	**<.001**	1.90	(1.49, 2.41)	**<.001**
46-65			**.005**			**<.001**			**<.001**
Continous Log-SII	1.21	(1.08, 1.35)	**.001**	1.24	(1.10, 1.39)	**<.001**	1.21	(1.07, 1.37)	**.002**
Quartile 1	Ref.			Ref.			Ref.		
Quartile 2	1.35	(1.06, 1.72)	**.015**	1.33	(1.05, 1.70)	**.021**	1.33	(1.02, 1.73)	**.035**
Quartile 3	1.22	(0.98, 1.51)	.076	1.24	(0.99, 1.55)	.057	1.24	(0.97, 1.58)	.087
Quartile 4	1.49	(1.19, 1.87)	**.001**	1.54	(1.22, 1.94)	**<.001**	1.48	(1.15, 1.91)	**.003**
>65			.342			.397			**<.001**
Continous Log-SII	1.11	(0.97, 1.26)	.126	1.10	(0.97, 1.26)	.145	1.08	(0.95, 1.23)	.248
Quartile 1	Ref.			Ref.			Ref.		
Quartile 2	1.32	(0.96, 1.81)	.087	1.32	(0.96, 1.81)	.090	1.23	(0.88, 1.72)	.232
Quartile 3	1.11	(0.84, 1.45)	.470	1.10	(0.83, 1.45)	.513	1.07	(0.80, 1.45)	.641
Quartile 4	1.23	(0.94, 1.62)	.135	1.23	(0.93, 1.63)	.152	1.19	(0.89, 1.61)	.243

The crude model did not adjust for covariates. Model 1 was adjusted for sex, age, and race. Model 2 was adjusted for sex, age, race, educational level, poverty income ratio, smoking status, alcohol consumption, serum creatinine, urine albumin to creatinine ratio, and glycated hemoglobin. Bold values indicate statistical significance (*P* < .05).

Abbreviations: CI, confidence interval; MetS, metabolic syndrome; OR, odds ratio; SII, systemic immune-inflammation index.

### Sensitivity Analysis

To enhance the robustness and representativeness of our study, a multiple imputation study was performed on datasets with missing confounder information (Table 4). Employing the multiple imputation by chained equations technique, we interpolated data into 20 groups based on age, sex, and ethnicity. This method, independent of data distribution, leverages the inherent characteristics of the dataset alongside suitable regression equations for imputation. It facilitates the accounting of missing data on variables including the family PIR, level of education, alcohol consumption status, smoking habits, Scr, the ratio of urine albumin to creatinine, glycated hemoglobin, as well as histories of hypertension and cancer. After imputation, analyses incorporating weighted multivariable logistic regression were executed before and after imputation according to model 2. Sensitivity analyses postimputation revealed a pronounced and statistically significant association between MetS and elevated levels of log2-SII across the total population, both as a categorized and as a continuous variable (OR = 1.21; 95% CI, 1.15-1.27; *P* < .001). Upon categorization of SII, the positive connection trend between SII levels and MetS prevalence persisted across all groups, with significant associations noted in all 3 models. In comparison with Q1, the risk of MetS in Q4 escalated by 53% in the imputed model (OR = 1.53; 95%CI, 1.37-1.72; *P* < .001).

**Table 4.** Association of SII with MetS before and after imputation

Age	Complete case			Multiple imputation		
	OR	(95% CI)	*P*-value	OR	(95% CI)	*P*-value
Overall			**<.001**			**<.001**
Continous Log-SII	1.25	(1.17, 1.33)	**<.001**	1.21	(1.15, 1.27)	**<.001**
Quartile 1	Ref.			Ref.		
Quartile 2	1.36	(1.18, 1.57)	**<.001**	1.26	(1.11, 1.43)	**<.001**
Quartile 3	1.29	(1.13, 1.48)	**<.001**	1.33	(1.18, 1.50)	**<.001**
Quartile 4	1.56	(1.37, 1.79)	**<.001**	1.53	(1.37, 1.72)	**<.001**
18-30			**<.001**			**<.001**
Continous Log-SII	1.47	(1.13, 1.90)	**.005**	1.52	(1.27, 1.81)	**<.001**
Quartile 1	Ref.			Ref.		
Quartile 2	1.63	(1.03, 2.59)	**.037**	1.53	(1.03, 2.25)	**.034**
Quartile 3	1.83	(1.15, 2.91)	**.011**	2.07	(1.39, 3.07)	**<.001**
Quartile 4	2.15	(1.28, 3.62)	**.004**	2.42	(1.61, 3.63)	**<.001**
31-45			**<.001**			**<.001**
Continous Log-SII	1.44	(1.26, 1.65)	**<.001**	1.39	(1.25, 1.56)	**<.001**
Quartile 1	Ref.			Ref.		
Quartile 2	1.34	(1.05, 1.71)	**.017**	1.33	(1.06, 1.67)	**.014**
Quartile 3	1.36	(1.06, 1.73)	**.015**	1.30	(1.05, 1.62)	**.017**
Quartile 4	1.90	(1.49, 2.41)	**<.001**	1.99	(1.59, 2.49)	**<.001**
46-65			**<.001**			**<.001**
Continous Log-SII	1.21	(1.07, 1.37)	**.002**	1.18	(1.07, 1.30)	**.001**
Quartile 1	Ref.			Ref.		
Quartile 2	1.33	(1.02, 1.73)	**.035**	1.22	(0.95, 1.55)	.113
Quartile 3	1.24	(0.97, 1.58)	.087	1.34	(1.07, 1.68)	**.012**
Quartile 4	1.48	(1.15, 1.91)	**.003**	1.43	(1.13, 1.80)	**.003**
>65			**<.001**			**<.001**
Continous Log-SII	1.08	(0.95, 1.23)	.248	1.04	(0.94, 1.14)	.491
Quartile 1	Ref.			Ref.		
Quartile 2	1.23	(0.88, 1.72)	.232	1.10	(0.80, 1.50)	.568
Quartile 3	1.07	(0.80, 1.45)	.641	1.04	(0.80, 1.35)	.781
Quartile 4	1.19	(0.89, 1.61)	.243	1.08	(0.85, 1.38)	.516

The crude model did not adjust for covariates. Model 1 was adjusted for sex, age, and race. Model 2 was adjusted for sex, age, race, educational level, poverty income ratio, smoking status, alcohol consumption, serum creatinine, urine albumin to creatinine ratio, and glycated hemoglobin. Bold values indicate statistical significance (*P* < .05).

Abbreviations: CI, confidence interval; MetS, metabolic syndrome; OR, odds ratio; SII, systemic immune-inflammation index.

Age-stratified sensitivity analysis observed that the incidence risk in the overall population and the 18 to 30 age group consistently increases with the elevation of the SII (OR = 1.52; 95% CI, 1.27-1.81; *P* < .001). In the young and middle-aged population (18-65 years), the highest incidence rates are found in the highest Q4 quartile, with significant statistical relevance across all groups. However, in the 31 to 45 age group, the level of increased risk of incidence in the Q2 and Q3 quartiles may vary before and after imputation, indicating that within the range of 321 < SII < 620, there may be an inconsistency in incidence rates with rising SII levels. Similarly, consistent with the postimputation model, the unimputed SII data still possess good predictive capability in the 18 to 45 age group, suggesting the stability of our results. For the population over 65 years old, the weighted multivariable logistic regression analysis indicated that treating SII as either a continuous or a categorical variable yielded results that were not statistically significant (*P* > .05) both before and after imputation.

### The Nonlinear Association Between the SII and MetS

**Figure 3.**
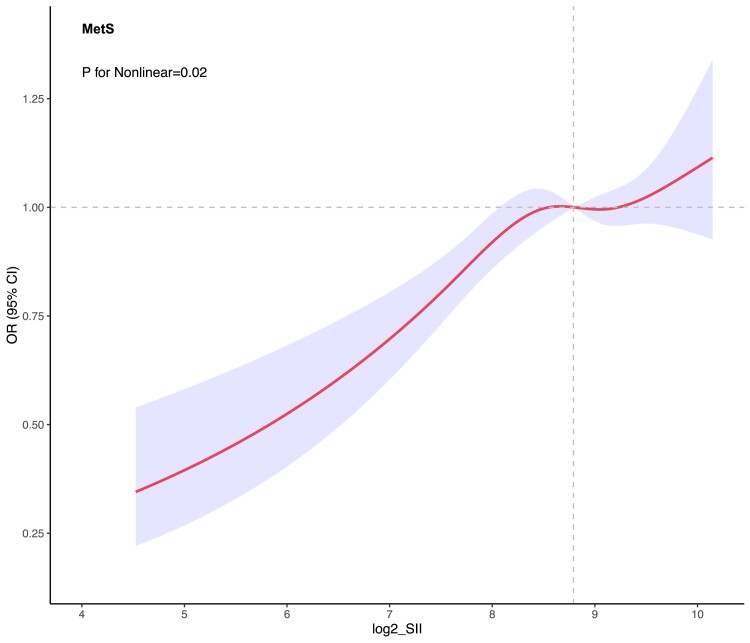
This figure depicts the nonlinear relationship between SII and MetS. The X-axis shows log-SII values, and the Y-axis shows the incidence of MetS. Shaded areas represent 95% confidence intervals. Abbreviations: MetS, metabolic syndrome; SSI, systemic immune-inflammation index.

Upon adjustment for a series of covariates, the RCS regression unveiled a notable nonlinear association between the log2-SII and MetS (*P* = .02, Fig. 1), manifesting as an inverted U-shaped curve (Fig. 3). The graphical representation delineated an ascending trend in MetS risk with increasing values of log-SII, which stabilized post an index value of 8.70, followed by a gradual deceleration. Notably, at the juncture where log2-SII reached 9.30, OR for MetS indicated a resurgence in the increasing trend.

**Figure 4.**
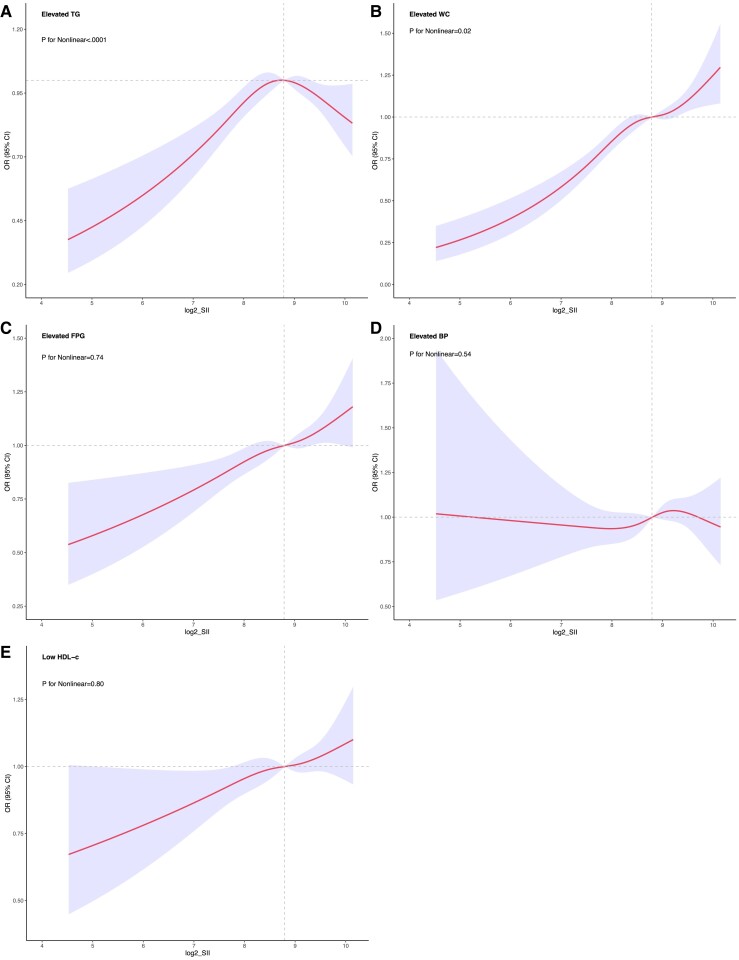
This figure shows the association between SII and components of MetS, including (A) elevated triglycerides, (B) elevated waist circumference, (C) elevated fasting glucose, (D) elevated blood pressure, and (E) low HDL-C. The X-axis shows log-SII values, and the Y-axis shows the incidence of MetS. Shaded areas represent 95% confidence intervals. Abbreviations: HDL-C, high-density lipoprotein cholesterol; MetS, metabolic syndrome; SSI, systemic immune-inflammation index.

When examining the components of MetS, log2-SII showed a positive correlation with FPG, low HDL-C, and high blood pressure. However, these did not fit a nonlinear relationship (*P* > .05 for all). RCS curves revealed a nonlinear correlation between log2-SII and both high TG and high WC among all participants (Fig. 4). Notably, beyond the inflection point of 8.71, OR for TG progressively decreased with an increase in log2-SII, indicating a negative correlation. In contrast, the risk of increased WC plateaued after a log2-SII of 8.70 and then gradually began to increase again.

### Nonlinear Relationships in Subgroup Correlations

**Figure 5.**
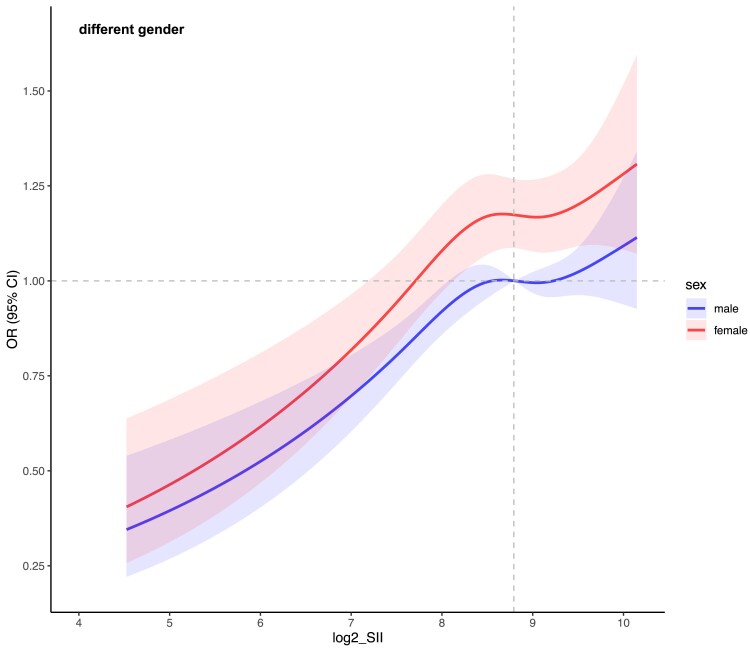
This figure illustrates the sex-specific nonlinear relationship between SII and MetS. The panels are divided by sex categories. The X-axis shows log-SII values, and the Y-axis shows the incidence of MetS. Shaded areas represent 95% confidence intervals. Abbreviations: MetS, metabolic syndrome; SSI, systemic immune-inflammation index.

**Figure 6.**
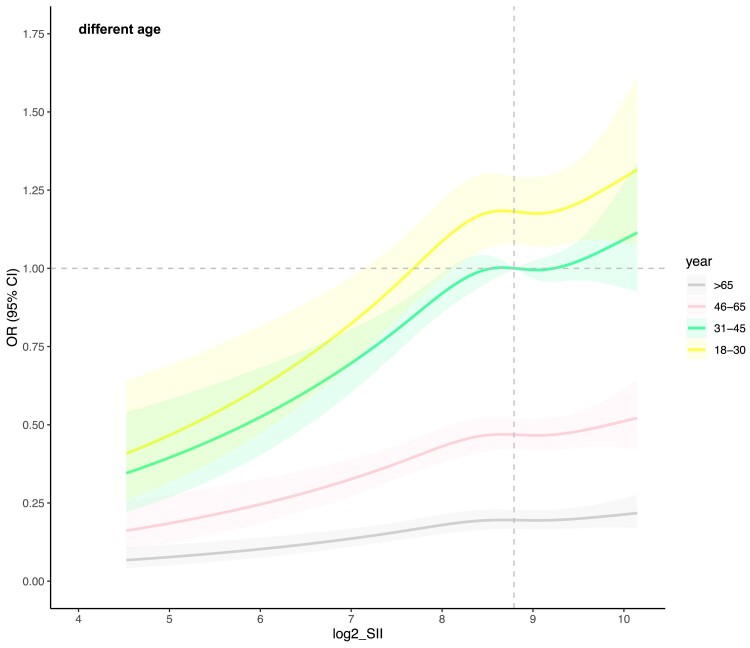
This figure illustrates the nonlinear association between SII and MetS across different age groups. The panels are divided by age categories. The X-axis shows log-SII values, and the Y-axis shows the incidence of MetS. Shaded areas represent 95% confidence intervals. Abbreviations: MetS, metabolic syndrome; SSI, systemic immune-inflammation index.

Upon stratification by sex and age for analysis (Figs. 5 and 6), it is observed that the nonlinear relationship trend between log2-SII and MetS remains consistent across all subpopulations. Within the age-stratified groups, individuals aged 18 to 30 exhibit a notably higher sensitivity in the connection between log2-SII and the risk of MetS incidence compared to other age groups, with the overall population risk plateauing as log2-SII increases beyond 8.71 (equivalent to SII = 418). In the sex-stratified groups, the association appears stronger in females than in males, indicating a differential impact of log2-SII on MetS risk between genders.

## Discussion

The balance between immune response and metabolic regulation is a critical homeostatic mechanism for the survival of organisms (36). Immune-inflammatory responses have long been recognized as key contributors to the pathogenesis of various diseases. Particularly in the global environment after the COVID-19 epidemic, the population's ability to resist infection has led to an enhanced immune response (37, 38), accompanied by subsequent metabolic adjustments aimed at preventing tissue damage and ensuring survival during the pandemic (39). Disruptions in metabolic functions also elevate the risk and mortality associated with cardiovascular diseases (40). Our study, drawing on large and representative national data spanning from 2001 to 2018, sought to investigate the link between inflammation levels and metabolic dysregulation. We discovered that the incidence of MetS is independently correlated with higher SII across the whole population. Even when confounding variables are taken into account, this association is still substantial. Moreover, we observed differences in the association across various age groups. The correlation was significant in the 18 to 65 age group, but it was not statistically significant in individuals over 65. In the 18 to 30 age cohort, the sensitivity of SII as a risk factor for predicting MetS is higher than in other age groups. This suggests that SII could be utilized for opportunistic screening of adverse metabolic factors in the youth population. Several guidelines highly value and recommend reasonable screening of these risk factors when visiting primary healthcare units (41). The association was analyzed using multiple imputation sensitivity analysis to minimize potential bias. Importantly, the measurement of the SII and the diagnosis of MetS are readily accessible within the primary healthcare system, covering a wide range of the population. Therefore, we recommend prioritizing the screening of high-risk populations in primary healthcare settings. Developing evidence-based treatment guidelines is essential to control excessive immune-inflammatory responses and metabolic dysregulation. This also provides evidence for further understanding the variations in inflammatory responses among different age groups in clinical or basic research.

The development of MetS is inseparable from a persistent, low-grade inflammatory condition (42). Due to the higher baseline level of inflammation, individuals with metabolic diseases have a higher probability and greater extent of damage from reaching pathological levels of inflammation during cytokine storms (43). This predisposes them to a greater risk of severe infection complications compared to metabolically healthy individuals prior to infection (39). Therefore, we believe that the proinflammatory state should also be included as a risk factor in the diagnostic criteria for MetS. Indeed, the inclusion of a proinflammatory state in the diagnostic criteria remains controversial. The ability of MetS to identify those at high risk for diabetes mellitus and cardiovascular illnesses is its most significant feature (4). Numerous studies have demonstrated notable differences in the occurrence and mortality risks of diabetes and cardiovascular diseases between subgroups with MetS and those without (44, 45). As mentioned earlier, increased inflammation is strongly correlated with a worse prognosis for metabolic and cardiovascular disorders. Large-scale clinical data exploring the mechanisms of inflammation and its association with disease incidence may support the establishment of this diagnostic criterion.

SII and MetS have been consistently correlated in recent investigations (46, 47). Our study additionally discovered that this association is more pronounced in the middle-aged and younger populations, whereas it was not statistically significant among the elderly. This demonstrates that SII can serve as an inflammatory indicator reflecting systemic inflammation in the middle-aged and young population. Inflammatory factors in young people are precisely and strictly regulated at low levels (48). After injury or infection, inflammatory factors rapidly increase in response and produce acute inflammatory reactions with self-limitation. Compared to the middle-aged and young populations, the elderly population exhibits a diminished capacity to resolve inflammation, resulting in the long-term production of proinflammatory cytokines and chemokines and the persistent infiltration of white blood cells into the tissue (49, 50). This condition, known as age-related inflammation, is defined by a long-term, low-grade aseptic inflammation that becomes apparent as people age. Therefore, even in healthy elderly populations, low to moderate levels of inflammatory factors are present. However, their expression remains lower compared to levels observed during acute infection states. The reasons for this phenomenon include chronic viral infections mentioned earlier, but also studies have shown that age-related visceral obesity, abnormal fat production, insulin resistance, lipotoxicity, and adipokine secretion can promote inflammatory responses (51). The difference in gut microbiota between the elderly and young populations also plays an important role in age-related inflammation (52, 53). Meanwhile, studies have observed that as age increases, levels of sex hormones decrease and levels of proinflammatory cytokines increase (54, 55). Other factors include lifestyle and environmental factors, lack of physical activity, dysbiosis of the microbiota, diet, psychological stress, and toxins. This indicates that the baseline inflammation level in elderly individuals is higher than that in younger individuals, which may account for the lack of correlation with disease-related predictive outcomes. In addition to the aforementioned factors, the high incidence rate of chronic diseases in the elderly and the anti-inflammatory effects of commonly used medications for cardiovascular and metabolic diseases may also contribute to false-negative results. In fact, due to the complexity of the inflammatory system, previous studies targeting a specific inflammatory factor in the same population may even exhibit contradictory situations (56). Differing from previous findings, our study shows that SII has limited predictive capability for MetS in the elderly. This suggests that monitoring immune cells at a single level may lose some predictive function for disease onset and progression. Further research is needed to explore more suitable inflammatory markers for the elderly population.

Due to their wide mobility, blood cells can reflect systemic inflammatory metabolism. The cascade reactions formed with other metabolic and immune cells can lead to pathological conditions and subsequently affect the body. In chronic inflammation, neutrophils can promote platelet production by secreting S100A8/A9, leading to a prethrombotic state characterized by neutrophil-platelet aggregates. This further increases inflammatory factors and enhances the migration and adhesion of neutrophils. Furthermore, neutrophils engage with adaptive immune cells, facilitating the onset and progression of chronic inflammation (57). Among diabetic patients, the adhesion of neutrophils to adipocytes contributes to the expression of chemotactic molecules and the infiltration of macrophages. This amplifies adipose tissue inflammation, leading to the incidence of insulin resistance (58). Similarly, monocytes, platelets, and white blood cells play crucial roles in stimulating the production and secretion of proinflammatory cytokines. They may accelerate the course of the disease by causing the production of receptors or other components that receive more inflammatory signals (59, 60). Research has found that there is an interdependent relationship between elevated platelet count and metabolic problems along with persistent low-grade inflammation (61, 62). Elevated white blood cell and platelet counts may serve as biomarkers for prethrombotic and proinflammatory conditions, leading to complications such as MetS and arterial thromboembolism (63). Therefore, an increased risk of thrombosis may be associated with higher SII levels, warranting additional follow-up studies to validate this association. Prior research has indicated that an increase in the number of MetS components, as well as an increase in MetS prevalence and risk, are correlated with platelet count (64, 65). Another study confirmed that alongside an increase in MetS components, there was an elevation in neutrophil and total white blood cell counts, accompanied by a significant decrease in lymphocyte counts (66). Studies have observed a decrease in lymphocyte counts in metabolic and cardiovascular diseases, along with an increased neutrophil-to-lymphocyte ratio in chronic diseases (67). Our research aligns with prior research, demonstrating that the occurrence of MetS increases with higher platelet and neutrophil counts and lower lymphocyte counts.

Our study possesses several strengths. First, it is the first research to specifically examine SII's function in MetS across various age groups. Ultimately, it was found that the sensitivity of the association is greater in middle-aged and young adults compared to the elderly. Second, our research employed a substantial, nationally representative cohort sample obtained through a cross-sectional study. This method enabled us to include a larger number of confounding factors in our data analysis, thereby ensuring the validity of our results. Thirdly, we performed multiple imputations for missing data to ensure the validity and reliability of our findings.

Nevertheless, this study also exhibits several limitations. A limitation that merits elaboration is we were unable to include all variables influencing the SII. Recent studies find that physical activity may directly or indirectly affect SII levels (68, 69). However, the classification standards for physical activity in the database changed between 2001 and 2018, which prevented us from generating a unified dataset on physical activity. Moreover, sarcopenia, a central comorbidity associated with age and metabolic syndrome (70), should not be overlooked in the middle-aged and younger population (71). Existing research consistently demonstrates that SII is elevated in patients with sarcopenia, which is closely linked to the development of obesity and adverse disease prognosis (72-76). High levels of SII have also been confirmed to be associated with the occurrence of sarcopenia in adults from middle-aged to younger populations (77). Whether this process mediates the development and progression of metabolic syndrome remains to be further verified. However, due to the lack of data, our study was unable to thoroughly investigate the interrelationships among these factors, which represents a limitation of our experiment. Second, although the SII is a dynamically changing numerical value, the nature of database sampling restricts us to extracting data from a single time point for analysis. Additionally, in practical analysis, we often exclude participants with incomplete data and outliers, leading to selection bias. Third, cross-sectional surveys hinder the establishment of a causal link between SII and MetS. Lastly, the inflammatory state may be influenced by medication use. While this may account for a small proportion in the younger population, the coexistence of underlying diseases and multiple illnesses in the elderly cannot be ignored. Therefore, the potential decrease in inflammation levels due to medication cannot be ruled out.

## Conclusion

Our results indicate a significant correlation between higher SII levels and the occurrence of MetS in middle-aged and young adults. The increase in SII levels is correlated with all 5 components of MetS, with WC being the most positively correlated component rise. The SII can serve as a direct and economical indicator for detecting MetS in individuals aged 18 to 65. The varying predictive value of the SII across different age groups warrants further investigation.

## Data Availability

The data analyzed in this study is publicly accessible through the National Center for Health Statistics website. For more information, please visit: https://www.cdc.gov/nchs/nhanes/.
