# Correction to: “Age Difference in the Connection Between Systemic Inflammatory Response and Metabolic Syndrome”

**DOI:** 10.1210/clinem/dgae869

**Published:** 2024-12-17

In the above-named article by Wei H, Xu D, Chen J, Yu H, Zhang X, Liu Z, Liu C, and Guo Y (*J Clin Endo Metab*. 2024; doi: 10.1210/clinem/dgae669), there was an error in the affiliations.

In the originally published article, in the affiliations, affiliation 1 read, “Department of General Practice, Zhongshan Hospital, Fudan University, Shanghai, 200030, China.” In the corrected article, affiliation 1 now reads, “Department of General Practice, Qilu Hospital of Shandong University, Jinan, 250012, China.” Because the original affiliation 2 is the same as the corrected affiliation 1, in the corrected article, affiliation 3 has been removed, and affiliation 2 now reads “School of General Practice and Continuing Education, Capital Medical University, Beijing, 100000, China.”

The article has been corrected online.

doi: 10.1210/clinem/dgae669

